# Synchronous Ileal Metastasis from Pancreatic Ductal Adenocarcinoma: Case Report and Narrative Review with Practical Diagnostic and Management Points

**DOI:** 10.3390/life15111684

**Published:** 2025-10-29

**Authors:** Tiberiu Stefăniță Țenea Cojan, Valeriu Șurlin, Stelian-Stefaniță Mogoantă, Nicolae-Dragoș Mărgăritescu, Daniel-Cosmin Caragea, Ioana-Alexia Țenea Cojan, Valentina Căluianu, Marius Cristian Marinaș, Gabriel Florin Răzvan Mogoș, Liviu Vasile, Laurențiu Augustus Barbu

**Affiliations:** 1 Department of Surgery, Railway Clinical Hospital Craiova, University of Medicine and Pharmacy of Craiova, 2 Petru Rares Street, 200349 Craiova, Romania; tiberiu.tenea@umfcv.ro (T.S.Ț.C.); valentina.andronache@yahoo.com (V.C.); laurentiu.barbu@umfcv.ro (L.A.B.); 2 Department of Surgery, Emergency County Hospital, University of Medicine and Pharmacy of Craiova, 2 Petru Rares Street, 200349 Craiova, Romania; vsurlin@gmail.com (V.Ș.); ssmogo@yahoo.com (S.-S.M.); dmargaritescu@yahoo.com (N.-D.M.); vliviu777@yahoo.com (L.V.); 3 Department of Internal Medicine, University of Medicine and Pharmacy of Craiova, 200349 Craiova, Romania; daniel.caragea@umfcv.ro; 4 Faculty of Medicine, University of Medicine and Pharmacy of Craiova, 200349 Craiova, Romania; teneaioana6@gmail.com; 5 Department of Obstetrics and Gynecology, Emergency County Hospital, University of Medicine and Pharmacy of Craiova, 2 Petru Rares Street, 200349 Craiova, Romania; cristian.marinas@umfcv.ro

**Keywords:** pancreatic ductal adenocarcinoma, synchronous metastasis, ileal obstruction, intestinal metastasis, immunohistochemistry, p53, CK7, CA19-9, palliative surgery

## Abstract

**Background**: Pancreatic ductal adenocarcinoma (PDAC) is an aggressive malignancy with poor prognosis, most frequently metastasizing to the liver, peritoneum, and lungs. Intestinal metastases are exceptionally rare and easily misinterpreted as primary small-bowel tumors, typically presenting with acute complications such as obstruction, perforation, or bleeding. **Methods**: We combined a detailed case description with a narrative literature review. PubMed/MEDLINE and Embase (2000–2025) were searched for case reports and case series describing intestinal metastases from PDAC with histopathological and immunohistochemical confirmation. **Case presentation**: We report a female patient presenting with acute intestinal obstruction caused by a synchronous ileal metastasis from PDAC. Imaging revealed an ileal stenosing lesion and a pancreatic body mass. An exploratory laparotomy identified a 3 cm transmural ileal tumor with additional serosal nodules. Histopathology confirmed a moderately differentiated adenocarcinoma. Immunohistochemistry supported pancreatic origin (CK7+, CA19-9+, faint CDX2), with mutant-type p53 positivity, ultra-low HER2/Neu expression, and a Ki-67 index of ~50%. The patient underwent segmental enterectomy with terminal ileostomy, followed by systemic therapy. **Conclusions**: This represents an exceptional and rare clinical finding rather than a presentation from which broad conclusions can be drawn. Histopathological and immunohistochemical analysis supported pancreatic origin and helped avoid misclassification as a primary intestinal neoplasm. It underscores the importance of careful clinicopathological correlation and multidisciplinary evaluation in atypical metastatic scenarios, while illustrating how surgery can provide symptom control and enable systemic therapy. Given its rarity, these observations should be interpreted with caution and regarded as descriptive rather than generalizable.

## 1. Introduction

Pancreatic ductal adenocarcinoma (PDAC) is a highly aggressive gastrointestinal malignancy, typically diagnosed at an advanced stage, with limited therapeutic options and poor survival outcomes. The 5-year survival rate remains below 10% overall and under 3% in metastatic disease. The liver, peritoneum, and lungs represent the most common metastatic sites, and systemic therapy remains the standard of care according to current ESMO and ASCO guidelines [1,2,3,4,5,6,7].

In contrast, small bowel metastases from PDAC are exceptionally rare, with only isolated reports in the literature [8,9,10,11]. When present, they often manifest through acute complications such as obstruction or bleeding, and synchronous presentations are particularly uncommon. We present a case of synchronous ileal metastasis causing acute small bowel obstruction, underscoring the diagnostic challenges of atypical metastatic patterns and the critical role of timely surgical palliation to enable systemic therapy.

## 2. Case Presentation

### 2.1. Patient Information

A female patient, previously diagnosed by imaging with a pancreatic tumor, was admitted to the Department of Surgery, Railway Clinical Hospital Craiova. Her medical history was significant for arterial hypertension and dyslipidemia. She presented with diffuse abdominal pain, absence of intestinal transit for feces and gas, and fecaloid vomiting with onset 2 days prior to admission.

### 2.2. Clinical Findings

On examination, the abdomen was mobile with respiratory movements, diffusely tender on palpation, distended in volume, and showed diffuse muscular guarding. Abdominal resonance was absent.

### 2.3. Timeline

A structured timeline of the patient’s clinical course, diagnostic workup, surgical intervention, and postoperative recovery is summarized below (Table 1).

### 2.4. Diagnostic Assessment

**Abdominal radiography:** No pneumoperitoneum; hydro-aerial levels in the mesogastrium.

**Ultrasonography:** Dilated intestinal loops with liquid content and hypermotility; no abdominopelvic free fluid.

**Figure 1:**
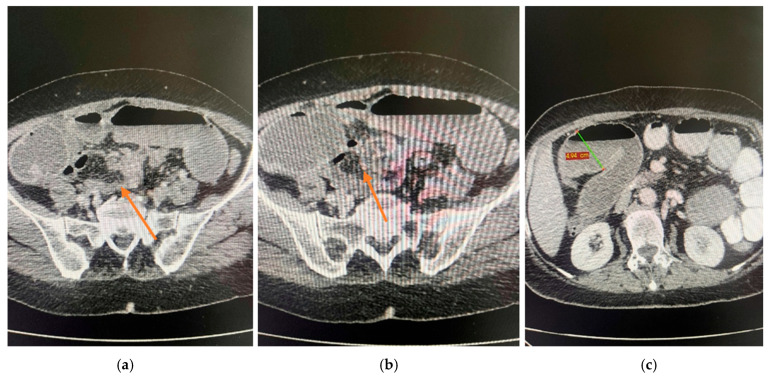
CT examination of the ileum. (**a**) Focal irregular parietal thickening (orange arrow). (**b**) Stenosing lesion (orange arrow). (**c**) Upstream intestinal dilatation (green measurement line).

**Figure 2:**
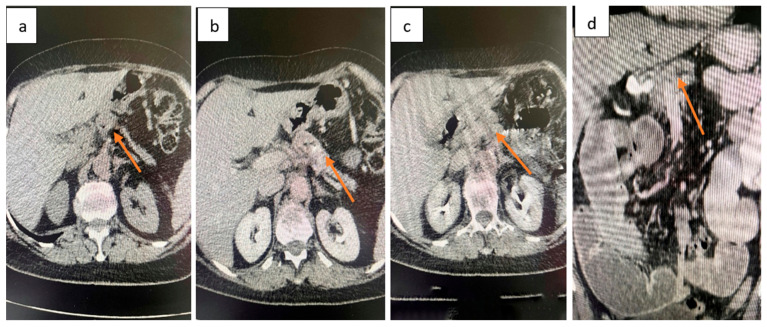
Contrast-enhanced abdominal CT. At the pancreatic body, a spontaneously isodense lesion with discrete iodophilia was identified, measuring 3.2 cm in axial diameter, without ductal dilatation (orange arrow). (**a**) Non-contrast phase. (**b**) Arterial phase. (**c**) Venous phase. (**d**) Delayed phase.

**CT abdomen and pelvis:** Focal, irregular parietal thickening with stenosing features located in the ileum, producing upstream intestinal distension with fluid content and hydro-aerial levels, without pneumoperitoneum (Figure 1). At the pancreatic body, a spontaneously isodense lesion with discrete iodophilia was identified, measuring 3.2 cm in axial diameter, without ductal dilatation (Figure 2). Given the acute obstructive presentation and the patient’s overall clinical status, EUS-FNB of the pancreatic mass was not performed preoperatively. This decision was agreed upon in a multidisciplinary tumor board, with the plan to reassess the lesion at a later stage for histological confirmation.

Laboratory investigations upon admission demonstrated leukocytosis with neutrophilia, indicating an acute inflammatory response. Coagulation studies were at the upper normal limit, while fibrinogen and ESR were markedly elevated, consistent with systemic inflammation. Renal function was preserved. Tumor markers showed a normal CEA but a mildly elevated CA 19-9, supporting possible pancreatic involvement (Table 2).

### 2.5. Therapeutic Intervention

**Figure 3:**
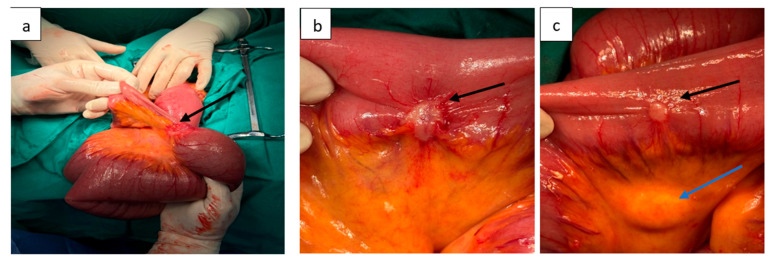
Intraoperative findings. (**a**) Stenosing ileal tumor (black arrow) with markedly dilated small bowel loops upstream and collapsed loops downstream. (**b**) Serosal tumor measuring 1.5 cm (black arrow). (**c**) Serosal tumor of 1 cm (black arrow) associated with intestinal lymphadenopathy (blue arrow).

Exploratory laparotomy revealed multiple dilated small bowel loops (8–10 cm in diameter, with thin walls). Approximately 50 cm proximal to the ileocecal valve, a 3 cm stenosing tumor extending beyond the serosa was identified. Two additional non-stenosing serosal nodules (1 cm and 1.5 cm, respectively) were also found upstream of the stenosis. In the corporeocaudal region of the pancreas, a firm, fixed mass measuring 5–5.5 cm was palpated, adherent posteriorly (Figure 3). The stomach and duodenum were markedly distended.

A segmental enterectomy was performed with terminal ileostomy in a “double-barrel” configuration.

### 2.6. Follow-Up and Outcomes

The postoperative course was favorable. The patient tolerated oral intake, had a functional ileostomy, and was discharged on postoperative day 10 in good general condition. She was subsequently referred to the oncology department and initiated systemic oncological treatment.

### 2.7. Histopathological Findings

**Figure 4:**
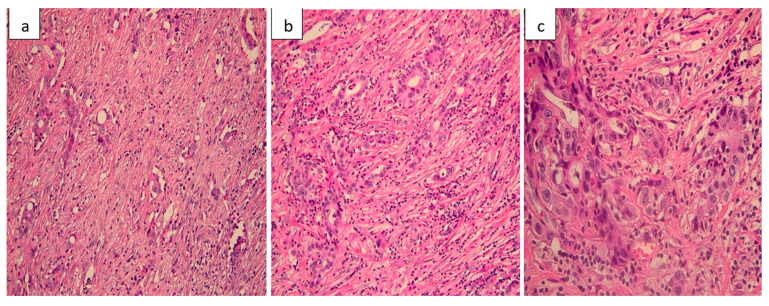
Moderately differentiated pancreatic adenocarcinoma. (**a**) Tumoral ducts with cuboidal cells, polymorphous nuclei, and frequent mitoses (H&E, 200×). (**b**) Cribriform structures and solid nests with desmoplastic stroma (H&E, 200×). (**c**) High-power view showing marked atypia and numerous mitotic figures (H&E, 400×).

**Figure 5:**
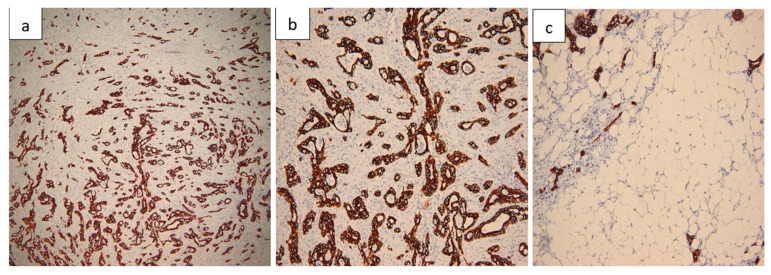
CK7 immunostaining. (**a**) Tubular pattern with complex architecture and isolated tumor cells at the invasion front (40×). (**b**) CK7 positivity highlighting the architectural complexity (100×). (**c**) Peripancreatic fat invasion on CK7 stain (100×).

**Figure 6:**
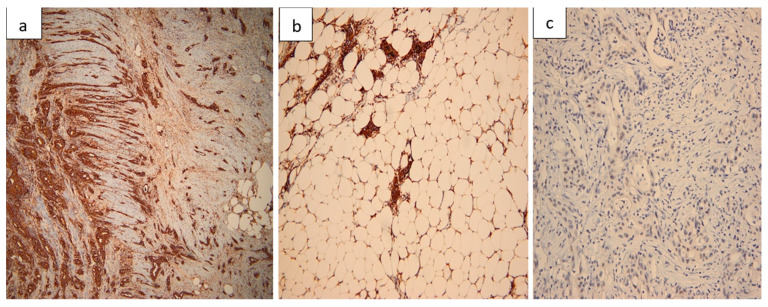
CA19-9/CDX2 immunoprofile. (**a**) CA19-9 positivity showing radial invasion in the muscularis propria of the duodenum (40×). (**b**) Peripancreatic fat invasion highlighted on CA19-9 stain (100×). (**c**) Very faint nuclear positivity for CDX2 in tumor cells (100×).

**Figure 7:**
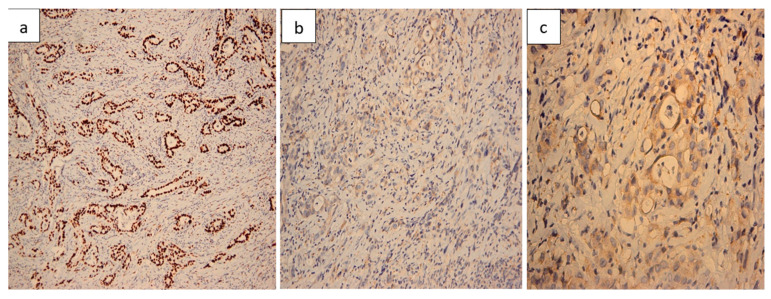
p53 and HER2/Neu immunoprofile. (**a**) Strong and diffuse mutant-type p53 positivity in tumor cells, with lighter wild-type staining in stromal cells (100×). (**b**) HER2/Neu ultra-low expression, with very faint membranous staining (20×). (**c**) Faint, discontinuous HER2/Neu immunostaining in scattered tumor cells (“hot spot”) (40×).

**Figure 8:**
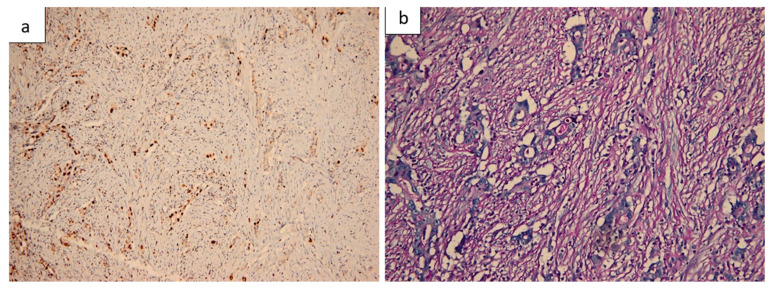
Ki-67 and PAS staining. (**a**) Ki-67 immunostaining showing a proliferation index of ~50% in tumor cells (100×). (**b**) Scant mucinous secretion in tubular lumina and within some tumor cells, highlighted by PAS staining (200×).

Histopathological evaluation of the resected small bowel segment revealed a moderately differentiated adenocarcinoma, measuring 3 cm, with transmural infiltration and extension beyond the serosa (Figure 4). Immunohistochemical staining demonstrated CK7 (Figure 5) and CA19-9 positivity, with only faint CDX2 expression (Figure 6), consistent with a pancreatic origin. Tumor cells exhibited strong and diffuse mutant-type p53 positivity (Figure 7a), ultra-low HER2/Neu expression (Figure 7b,c), and a Ki-67 proliferation index of approximately 50% (Figure 8a). Scant mucinous secretion in tubular lumina and within some tumor cells was highlighted by PAS staining (Figure 8b). MMR protein expression was preserved, indicating a low probability of high microsatellite instability (MSI-H). Taken together, these findings supported the diagnosis of intestinal metastasis from a moderately differentiated pancreatic adenocarcinoma. We expanded the IHC panel to differentiate from a primary colonic or ileal tumor (CK20, SATB2, MUC1, SMAD4, CK19). The resulting profile (CK7+, CA19-9+, CK20−, SATB2−, MUC1+, SMAD4 loss, very faint CDX2) supports a pancreatic origin; however, in the absence of a pancreatic mass biopsy, the conclusion remains qualified as probable.

## 3. Materials and Methods

### 3.1. Literature Search and Selection

A narrative literature review was performed in PubMed/MEDLINE and Embase databases, covering the period 2000–2025. The following search terms were applied: *“pancreatic ductal adenocarcinoma” OR “PDAC” AND “intestinal metastasis” OR “small bowel metastasis” OR “colonic metastasis”*. In addition, the reference lists of relevant articles were screened manually to identify further eligible studies.

Inclusion criteria were case reports or case series reporting histologically confirmed intestinal metastases from PDAC. Studies were excluded if they described direct contiguous invasion without evidence of metastatic spread, lacked immunohistochemical confirmation, or were published in languages other than English.

### 3.2. Limitations

The evidence base is restricted to isolated case reports, which are inherently prone to publication bias. Further heterogeneity results from variability in immunohistochemical panels and occasional diagnostic uncertainty in differentiating true metastases from direct invasion. Consequently, quantitative synthesis was not feasible, and the results were analyzed qualitatively.

## 4. Discussion

More than half of patients with pancreatic ductal adenocarcinoma (PDAC) present with metastatic disease at diagnosis, most frequently involving the liver, peritoneum, and lungs [1,2]. Reports of small-bowel metastases are limited to isolated case descriptions, and no reliable incidence data are available [8,9,10].

### 4.1. Epidemiology and Mechanisms of Spread

Pancreatic ductal adenocarcinoma (PDAC) is one of the most lethal gastrointestinal malignancies and currently the seventh leading cause of cancer-related death worldwide, projected to rank second by 2030 [1,2]. Despite therapeutic progress, the 5-year survival remains 6–10%, and below 3% in metastatic disease [1]. Over half of patients present with advanced disease, with the liver (60–70%), peritoneum (20–40%), and lungs (10–20%) as the predominant metastatic sites [2,3]. Gastrointestinal involvement is exceptionally rare and reported almost exclusively as isolated case reports, with colonic and small-bowel metastases documented by immunohistochemical confirmation of pancreatic origin [8,9,10].

Three main mechanisms are proposed to explain these rare metastatic patterns. Hematogenous dissemination may account for distant intestinal lesions via tumor thrombi in the portal or systemic circulation, often without peritoneal involvement [1,3,11]. Lymphatic spread through mesenteric or retroperitoneal pathways can lead to skip metastases in the absence of dominant hepatic disease [3,9]. Finally, peritoneal seeding through transcoelomic spread allows exfoliated tumor cells to implant on the bowel surface and invade the wall, particularly in carcinomatosis or ascites, explaining some synchronous obstructive presentations [10,11].

### 4.2. Correlation with Reported Cases

**Figure 9:**
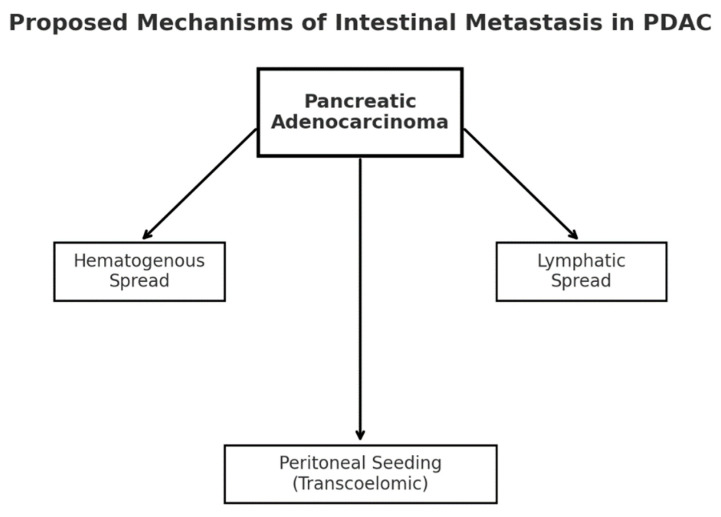
Proposed mechanisms of intestinal metastasis in PDAC. Legend: Three main routes have been proposed—(1) hematogenous dissemination to distant bowel, (2) lymphatic spread via mesenteric/retroperitoneal channels, and (3) peritoneal seeding with secondary bowel wall invasion [1,3,10].

Published cases illustrate these mechanisms (Figure 9). Metachronous jejunal and colonic metastases occurring after pancreaticoduodenectomy presented with obstruction and fever, requiring emergency resection; the patient died within seven months, consistent with aggressive disease and hematogenous or lymphatic spread [9]. A jejunal metastasis diagnosed five years after curative resection, confirmed by IHC (CK7+, CK20−), was resected with favorable recovery, and the absence of peritoneal disease suggested hematogenous or lymphatic dissemination [8]. A synchronous sigmoid metastasis with pancreatic-type IHC (CK7+, CK20−) was reported as an unusual metastatic pattern, with hematogenous, lymphatic, and transcoelomic routes considered plausible [11].

### 4.3. Clinical Presentation and Diagnostic Work-Up

The clinical presentation of intestinal metastases from pancreatic ductal adenocarcinoma (PDAC) is nonspecific and often leads to delayed diagnosis. Typical symptoms include abdominal pain, nausea, vomiting, altered bowel habits, and signs of subocclusion or obstruction [9,10]. Occult gastrointestinal bleeding with anemia may be the initial manifestation [8], while hematemesis, melena, and iron-deficiency anemia can mimic other acute gastrointestinal conditions [12]. Obstructive symptoms may resemble mesenteric lymphatic cysts or appendiceal tumors [13,14]. Rarely, patients present with perforation and peritonitis requiring emergency surgery [10], and differential diagnoses should include colonic, rectal, or retroperitoneal pathologies [15]. Because of its rarity, intestinal involvement is often mistaken for a primary gastrointestinal tumor, underscoring the importance of histopathological confirmation.

The diagnosis is challenging and relies on appropriate imaging and histopathology. Contrast-enhanced CT is the first-line investigation to assess obstruction and metastatic spread [1], but sensitivity decreases for small or early lesions. Careful review of multiphasic CT is important in pancreatic tail primaries near the splenic hilum or flexure, where direct invasion may mimic metastasis. PET-CT can identify hypermetabolic bowel lesions when CT or endoscopy are inconclusive, as shown in a reported case of jejunal recurrence [8]. Endoscopy or capsule endoscopy may detect intraluminal lesions but have limited value for extramural disease. A definitive diagnosis requires histopathological and immunohistochemical confirmation of pancreatic origin [1].

### 4.4. Histopathological and Immunohistochemical Evidence

True small-bowel metastases from pancreatic ductal adenocarcinoma (PDAC) must be differentiated from direct invasion of adjacent loops and from primary intestinal adenocarcinoma. Immunohistochemistry (IHC) plays a key role in this distinction; a CK7+/CK20−/CDX2− profile supports pancreatic origin, while CK20+/CDX2+/SATB2+ suggests a colorectal primary [8,11]. Neuroendocrine tumors (NETs) of the small intestine, another important differential diagnosis, are typically chromogranin A and synaptophysin positive and may present with obstruction, anemia, or abdominal pain [16]. Primary intestinal lymphomas can also mimic these presentations, often debuting with obstruction or upper gastrointestinal symptoms [17].

Histologically, PDAC metastases usually involve the subserosa or submucosa with preservation of the mucosa. Deep biopsies are recommended because superficial samples can mimic a colorectal phenotype overlying a pancreatic metastasis. A practical diagnostic profile includes CK7+/CK20−/CDX2−, often MUC1+, with possible SMAD4 loss, whereas colorectal primaries are typically CK20+/CDX2+/SATB2+ and CK7−. Additional markers such as CK19 and CA19-9 may provide further support. Strong and diffuse p53 positivity, as in our case, is consistent with tumor progression and aids in distinguishing PDAC metastases [18].

Published reports describe identical IHC profiles in pancreatic primaries and intestinal lesions, most commonly diffuse CK7 positivity with CK20 negativity, supporting the diagnosis of metastatic spread rather than synchronous primaries [8,11]. This confirmation often guides therapy, favoring palliative surgery followed by systemic treatment. When feasible, pancreatic mass biopsy with IHC comparison (±KRAS/SMAD4) is recommended to exclude a synchronous luminal primary. If biopsy is not possible, extended IHC on the metastasis, correlated with morphology and imaging, helps reduce misclassification, though the conclusion remains probabilistic. Because aberrant marker expression may occur, final diagnosis should integrate clinical, radiologic, and pathological data, and when necessary, additional molecular testing.

### 4.5. Molecular and Therapeutic Considerations

Molecular profiling complements histopathology and immunohistochemistry in pancreatic ductal adenocarcinoma (PDAC) by identifying clinically relevant alterations. KRAS mutations are present in approximately 95% of cases, with the G12R variant—around 15%—showing distinct signaling that may confer relative sensitivity to MEK inhibition, though this remains investigational [19,20,21]. A subset of tumors harbor DNA damage repair alterations (BRCA1, BRCA2, PALB2), associated with platinum sensitivity and potential response to PARP inhibitors. Rare MSI-high tumors may benefit from immune checkpoint blockade [22,23]. For this reason, baseline next-generation sequencing is increasingly recommended in advanced PDAC to identify actionable targets, in line with ESMO, NCCN, and ASCO guidelines [6,24,25].

Accurate classification of intestinal lesions—distinguishing metastasis from a primary tumor or direct invasion—is crucial for therapeutic planning. Surgery or endoscopic procedures are typically reserved for complications such as obstruction or bleeding, or for carefully selected oligometastatic cases, while systemic therapy remains the standard of care [26]. Surgical intervention is most often used for acute complications, with reported procedures including segmental bowel resection, bypass, or stoma formation, resulting in short-term symptom relief [8,9,10]. Endoscopic palliation with self-expanding metal stents can decompress obstructive colonic lesions and serve as a bridge to systemic treatment. Chemotherapy regimens include FOLFIRINOX for fit patients and gemcitabine plus nab-paclitaxel for those with lower performance status, often initiated or resumed after palliation [2,27]. Given the aggressive course of PDAC, multidisciplinary decision-making is essential to balance surgical risks, expected benefits, and the feasibility of continuing systemic therapy.

### 4.6. Practical Management Algorithm for Intestinal Metastasis from PDAC

**Step 1.** Clinical suspicion

Patient with pancreatic adenocarcinoma presenting with abdominal pain, nausea, vomiting, subocclusion/obstruction, anemia, or GI bleeding [9,10].

**Step 2.** Diagnostic work-up

CT scan → first-line to assess site of obstruction and metastases [1].

PET-CT → for occult or equivocal lesions [8].

Histopathology + IHC (CK7+, CK19+, CA19-9+, CK20−, CDX2−) → confirm pancreatic origin [11].

**Step 3.** Emergency/urgent management

Endoscopic palliation (self-expanding metal stents) → in selected colonic obstruction.

Surgical resection (segmental bowel resection ± anastomosis or bypass) → for obstruction, perforation, bleeding [10,28].

Re-resection may be considered in oligometastatic disease with good performance status [8].

**Step 4.** Post-palliation systemic therapy

Resume or initiate systemic treatment:

FOLFIRINOX (fit patients).

Gemcitabine + nab-paclitaxel (more common in reported cases, tolerable) [2,27].

**Step 5.** Multidisciplinary follow-up

Regular monitoring with imaging + CA19-9.

Focus on symptom relief, nutritional support, and maintaining chemotherapy eligibility.

### 4.7. Prognosis and Clinical Lessons

The prognosis of pancreatic ductal adenocarcinoma (PDAC) remains poor, with a 5-year survival of 9–10% across all stages and less than 3% in metastatic disease [1,2]. Median survival in advanced or unresectable stages is short, underscoring the need for individualized multimodal strategies [1]. Outcomes of intestinal metastases are rarely documented; Miyasaka et al. reported death seven months after emergency resection for jejunal and colonic involvement [9], whereas Tseng et al. described favorable recovery and ongoing systemic therapy after re-resection of a metachronous jejunal metastasis nearly five years post-pancreatic surgery [8].

Prognosis is influenced by several factors. Tumor burden is critical—patients with oligometastatic disease may benefit from selective resection, while those with disseminated disease have a significantly worse outcome [8,9]. Performance status, reflected by ECOG score and suitability for systemic therapy (FOLFIRINOX or gemcitabine plus nab-paclitaxel), strongly impacts survival [1,8]. In selected patients, re-resection of intestinal metastases may offer symptom control and enable continuation of treatment [8].

Although long-term survival is uncommon, palliative surgery can provide rapid symptom relief, prevent life-threatening complications, and allow systemic therapy to continue. Miyasaka et al. reported good postoperative recovery with normalization of fever and inflammatory markers and hospital discharge [9]. Similarly, Meng et al. described initiation of gemcitabine plus nab-paclitaxel following IHC confirmation of pancreatic origin in a case of colonic metastasis [11].

### 4.8. Reported Cases in the Literature

A review of the literature (Table 3) highlights the extreme rarity of intestinal metastases from PDAC, with fewer than 15 well-documented cases over the last decade. Most cases were reported as **single case reports**, reflecting the anecdotal nature of the available evidence.

Most patients with intestinal metastases from pancreatic ductal adenocarcinoma (PDAC) present with acute complications such as obstruction, abdominal pain, or perforation, while fewer are diagnosed incidentally during colonoscopy or imaging. Rare metachronous cases may occur years after pancreatic resection. The jejunum, ileum, and colon (sigmoid, transverse, rectum) are most often affected, with exceptional cases involving colonization of preexisting colorectal carcinoma, suggesting atypical spread [8,9,10,11].

Management is mainly palliative, typically involving emergency or symptom-driven segmental colectomy or enterectomy, with endoscopic stenting used selectively in colonic obstruction. Systemic chemotherapy (gemcitabine plus nab-paclitaxel or FOLFIRINOX) is often initiated or resumed after palliation. Prognosis remains poor, though re-resection may benefit carefully selected oligometastatic patients. In most cases, surgery provides symptom relief, improved quality of life, and enables continuation of systemic therapy [8,9,10,11].

### 4.9. Key Messages

Key practice points in diagnosing and managing intestinal metastases from PDAC are summarized in Table 4.

1. Intestinal metastases from PDAC are exceptional events, often presenting with acute surgical emergencies.
2. Surgical resection provides palliative benefit and may enable systemic therapy in selected patients.
3. The role of systemic chemotherapy is crucial, but evidence remains anecdotal.
4. Multidisciplinary evaluation is essential, as no standardized guidelines exist.

### 4.10. Review Synthesis and Future Directions

Available evidence indicates that intestinal metastases from pancreatic ductal adenocarcinoma (PDAC) are exceptionally rare and usually present with obstruction, bleeding, or acute abdomen. Because clinical and imaging findings are nonspecific, histopathological and immunohistochemical confirmation is crucial. Although mainly palliative, surgery provides symptom relief and preserves eligibility for systemic therapy, the mainstay of treatment. Recognizing this entity helps avoid misdiagnosis and supports timely management.

Current knowledge relies on isolated case reports and small series, with no standardized guidelines. Future research should focus on registries to clarify incidence, risk factors, and outcomes, and on systematic reporting to refine diagnosis. Consensus recommendations are needed to standardize palliation, integrate systemic therapy, and optimize follow-up. This case adds to the limited literature by documenting a rare synchronous intestinal metastasis with acute obstruction and highlighting the value of such reports for future clinical practice.

## 5. Conclusions

Synchronous ileal metastasis from PDAC represents an exceptional and rare clinical finding, rather than a typical presentation from which general conclusions can be drawn. Histopathological and immunohistochemical analysis supported a pancreatic origin and helped avoid misclassification as a primary intestinal neoplasm. Surgical palliation provided symptom control and allowed systemic therapy to be initiated. As pancreatic mass biopsy was not performed in the acute setting, the diagnosis should be regarded as probable and interpreted cautiously, in conjunction with clinical, imaging, and IHC findings. This highlights the importance of multidisciplinary evaluation and careful, case-by-case decision-making in unusual metastatic scenarios, as well as the value of reporting rare cases to support future evidence-building rather than to establish clinical standards.

## Figures and Tables

**Table 1:** Clinical Timeline of Patient Presentation and Management.

Day	Clinical Event
**Day 0**	Onset of diffuse abdominal pain, fecaloid vomiting, and absence of intestinal transit.
**Day 2**	Admission to hospital; clinical and imaging workup performed.
**Day 2 (evening)**	Decision for surgical intervention; exploratory laparotomy performed.
**Postoperative Days 1–3**	Gradual clinical improvement; ileostomy functional; oral intake initiated.
**Postoperative Days 4–9**	Continued recovery without complications.
**Postoperative Day 10**	Discharged home in good general condition.

**Table 2:** Laboratory tests upon admission.

Parameter	Result	Normal Range
**White blood cell count**	13.40 × 10^3^/μL	4–10 × 10^3^/μL
**Neutrophil proportion**	77.9%	40–70%
**Hemoglobin**	13.4 g/dL	12–16 g/dL
**Platelet count**	304 × 10^3^/μL	150–450 × 10^3^/μL
**Creatinine**	0.7 mg/dL	0.6–1.3 mg/dL
**Urea**	28.37 mg/dL	15–45 mg/dL
**INR**	1.21	0.8–1.2
**Prothrombin time**	14.3 s	11–15 s
**Fibrinogen**	1115 mg/dL	200–400 mg/dL
**C-reactive protein**	—	<0.5 mg/dL
**ESR**	55 mm/h	<20 mm/h
**CEA**	1.23 ng/mL	<5 ng/mL
**CA 19-9**	53.11 U/mL	<37 U/mL

**Table 3:** Reported cases of intestinal metastases from pancreatic ductal adenocarcinoma (PDAC).

Author/Year	Patient (Age/Sex)	Interval from Primary PDAC	Site of Metastasis	Presentation	Management	Outcome
**Fasano** [10]	Long-term survivor (M)	Synchronous	Small bowel	Acute abdomen	Emergency small bowel resection	First described intestinal metastasis in long-term PDAC survivor with mesothelioma
**Miyasaka** [9]	63/M	3 months post-pancreaticoduodenectomy	Jejunum + colon	Abdominal pain, fever, diarrhea	Emergency resection (jejunum + colon)	Death at 7 months due to relapse
**Nakaya** [27]	72/M	Synchronous	Colon (within preexisting carcinoma)	Bowel obstruction	Colectomy	Rare phenomenon of PDAC metastasis colonizing colon carcinoma
**Park** [28]	73/F	Synchronous	Transverse colon	Large bowel obstruction	Segmental colectomy	Palliative survival, <1 year
**Kahl** [29]	91/F	Synchronous	Sigmoid colon	Large bowel obstruction	Sigmoid colectomy	Died shortly after surgery
**Meng** [11]	65/M	Concomitant	Sigmoid colon	Colon lesion detected on work-up	Diagnosis confirmed by IHC; chemotherapy	Extremely rare metastatic route
**Ardalan** [30]	66/F	Synchronous	Sigmoid colon	Acute obstruction	Emergency left hemicolectomy + IHC; FOLFIRINOX	Poor tolerance, palliative course
**O’Sullivan** [24]	Cases (2)	Synchronous	Rectum/colon	Obstruction, GI symptoms	Surgical resection/supportive	Reported as very rare synchronous cases
**Tseng** [8]	56/F	~5 years post-pancreaticoduodenectomy	Jejunum	Anemia (Hb 8–9 g/dL), occult bleeding	Segmental jejunal resection	Uneventful recovery; continued systemic therapy
**Pacheco** [31]	68/M	Metachronous (interval not specified)	Sigmoid colon	Symptomatic metastasis, bowel obstruction	Surgical resection	Reported as the third symptomatic colonic metastasis from PDAC
**Present case (2025)**	F	Concomitant	Ileum	Acute intestinal obstruction	Emergency ileal resection + anastomosis	Favorable recovery; referred to oncology.

**Table 4:** Lessons learned/Practice points in diagnosing and managing intestinal metastases from PDAC.

Practice Point	Rationale
**When technically feasible, target submucosa**	Superficial mucosal biopsies may mimic a colorectal phenotype; submucosal sampling can improve diagnostic accuracy, but should not be considered mandatory
**Always run CK7/CK20/CDX2/SATB2 ± MUC1/SMAD4**	Distinguishes PDAC metastasis (CK7+/CDX2−/SATB2−) from primary intestinal adenocarcinoma.
**Re-discuss at MDT before major surgery**	Avoids inappropriate CRC-style resections and aligns care with systemic PDAC therapy. Avoiding inappropriate surgery in such rare scenarios is not based on a standardized algorithm, but rather on individualized, case-by-case multidisciplinary decision-making, aimed at aligning management with the underlying pancreatic primary.

Note: Adapted from published case reports and small series on intestinal metastases from PDAC [8,9,10,11,32,33,34,35,36,37,38,39].

## Data Availability

The data presented in this study are available on request from the corresponding author. The data are not publicly available due to patient confidentiality.
